# The Role of Veterinarians in Mass Casualty Disasters: A Continuing Need for Integration to Disaster Management

**DOI:** 10.3389/fpubh.2021.644654

**Published:** 2021-08-11

**Authors:** Lindsey S. Holmquist, James Patrick O'Neal, Ray E. Swienton, Curtis A. Harris

**Affiliations:** ^1^Institute for Disaster Management, College of Public Health, University of Georgia, Athens, GA, United States; ^2^Division of Emergency and Disaster Global Health, University of Texas Southwestern Medical Center at Dallas, Dallas, TX, United States; ^3^Department of Emergency Medicine, University of Texas Southwestern Medical Center at Dallas, Dallas, TX, United States; ^4^Institute for Disaster Management, College of Public Health, University of Georgia, Athens, GA, United States

**Keywords:** veterinarian, disasters, mass casualty, disaster management, disaster medical response

## Abstract

The need to prepare veterinarians to serve as part of the disaster medical response for mass casualty incidents has been recognized since at least the 1960's. The potential value of incorporating veterinarians for mass casualty disaster response has been noted by organizations throughout the world. Clinical veterinarians are highly trained medical professionals with access to equipment, medications, and treatment capabilities that can be leveraged in times of crisis. The ongoing threat of disasters with the current widespread healthcare access barriers requires the disaster management community to address the ethical constraints, training deficiencies and legal limitations for veterinary medical response to mass casualty disasters. An ethical imperative exists for veterinarians with translatable clinical skills to provide care to humans in the event of a mass casualty disaster with insufficient alternative traditional medical resources. Though this imperative exists, there is no established training mechanism to prepare veterinarians for the provision of emergency medical care to humans. In addition, the lack of clear guidance regarding what legal protections exist for voluntary responders persists as a barrier to rapid and effective response of veterinarians to mass casualty disasters. Measures need to be undertaken at all levels of government to address and remove the barriers. Failure to do so reduces potentially available medical resources available to an already strained medical system during mass casualty events.

## Introduction

Veterinarians are highly trained professionals with skills ranging from public health planning, food and water assessment, laboratory analysis, and a range of animal clinical specialties. In the United States, veterinarians' involvement in disaster management has been recognized in literature and policy, and those roles are continually evolving as the threat environment changes, technology develops, and the field of disaster management matures (1–9). In 1964, the Office of Civil Defense commissioned a Public Health Service report to identify the expanded and additional responsibilities veterinarians could assume to preserve human health after a disaster. In addition to animal care and public health roles, the report presented rationale and way forward for veterinarians to deliver medical care to humans following a disaster (10). Published literature from around the world echoes the roles of veterinarians in preserving human life and health in disasters (3, 11, 12). The American Veterinary Medical Association's policy “Addressing the Role of Veterinary Medicine in Human Health Care Following Catastrophes Involving Mass Human Casualty” formally recognizes the increasing role of veterinarians in disaster management and urges governments to address legal and political barriers to veterinarian involvement in disaster response. Published in 2010, this policy was intended to encourage collaboration between human health and veterinary assets “to further the nation's response readiness and create safer, more resilient communities.” (13). However, the question remains, “Has this been done?” Though the political, threat, and preparedness environments have changed since the 1964 report was published, the value of preparing veterinarians to provide medical care for humans following a disaster still remains. With appropriate training, guidance, and oversight, this skilled workforce would be a significant asset in any disaster response.

## Why Veterinarians?

The veterinary medical degree curriculum creates versatile professionals with a wide array of skills and broad knowledge base. In addition to public health courses covering food and water protection, basic epidemiology, and zoonotic disease, veterinarians receive instruction on a wide range of anatomies, physiologies, pathologies, and diagnostic and treatment modalities. After graduation, clinical veterinarians continue to hone and develop their diagnostic and treatment skills through the practice of veterinary medicine. As a testament to their professional flexibility, clinical veterinarians are accustomed to adjusting their assessment and treatment of patients based on species, size, and unique behavioral characteristics. To expand and adapt current knowledge to include humans in the range of animals for which veterinarians could be prepared to provide life-saving interventions is not outside the realm of reason. Veterinarians in clinical practice generally have access to diagnostic imaging capabilities, basic laboratory equipment, surgical suites, sterilization abilities, and pharmacy stocks that would allow them to assess, diagnose, and treat a number of conditions humans would face in a disaster scenario.

## Addressing the Challenges

### Ethical Challenges

Ethical challenges will arise if clear priorities for disaster response are not explicit and outlined in policy documents. The American Medical Association's Council on Ethical and Judicial Affairs included four criteria in their expectations of physicians to respond to disaster situations: 1. Degree of need, 2. Proximity to the need, 3. Capability to provide aid, and 4. Availability of other aid sources (14). Veterinarians possess the knowledge and skill to preserve human life and health (as highlighted in the AVMA's mass casualty policy) which satisfies the third criterion. When faced with disaster situations in which the other criteria are met, the failure to incorporate veterinarians in disaster medical planning can lead to complicated ethical dilemmas (15). For example, consider a rural setting in which a large organophosphate chemical spill occurs. Is there an ethical obligation of the local veterinarian to respond and provide atropine to the affected to increase the number of survivors? Does the ethical imperative of the veterinarian to respond increase with a corresponding decrease in the capacity of the existing medical capabilities?

Currently, veterinary assets are listed in policy recommendations as resources that can be called on if all other medical resources have been exhausted (16, 17). The threshold of “overwhelmed medical resources” is unclear and authority to supplement with veterinary resources is ill-defined. Waiting until the available medical resources are completely overwhelmed or exhausted is a dangerous proposal. The outcomes of failure to effectively integrate various community assets to disaster health care management can be seen in the inadequate responses to Hurricane Katrina in 2005 and the H1N1 pandemic influenza in 2009 (18). To address the root causes of these ineffective responses, health care coalitions were developed. Though individual health care coalitions vary in exactly which community assets are included, in general health care coalitions are “a group of health care organizations and public safety and public health partners that join forces for the common cause of making their communities safer, healthier, and more resilient” (18). The health care coalition is a logical integration point for veterinary assets to become more involved in disaster preparedness and response activities. Effective integration of veterinarians in all phases of disaster management would encourage appropriate resource allocation, outline expectations, build confidence, and strengthen professional relationships. Public acceptance of veterinarians providing disaster medical care may be accomplished through a marketing campaign carried out by state or local healthcare entities. The campaign could foster public confidence in the health care system to provide care in the event of a disaster and normalize the receipt of disaster care by veterinarians.

Unfortunately, contingency personnel are rarely included in healthcare disaster preparedness activities. Even in a recently released disaster medicine reference text, it is recommended that the healthcare system identify supplementary healthcare personnel (i.e., dentists, veterinarians, etc.) in the event of a disaster, but there is no mention of including these resources in drills and exercises (19). Responding personnel of all professions should know where to report and what conditions trigger their activation (20). The inherent nature of a “crisis standards of care” situation will raise ethical issues for all medical responders, but prior training for responding veterinarians on limited-resource clinical protocols or algorithms for human patients could improve response (21). In addition, the inclusion of veterinarians to disaster response training and exercises provides these professionals the opportunity to develop contingency plans for the care of their own animal patients should the need to respond to a human mass casualty disaster arise.

### Recommendations for Training

The 1964 report “The Role of the Veterinarian in National Disaster” recommends that, in addition to training on general disaster information and community disaster plans, training on diagnostic equipment, laboratory test interpretation, and pharmaceutical use for humans should be given by physicians to veterinarians (10). A recent study of private veterinary practitioners in Mississippi reported that just over 20% of respondents had any formal disaster training, but approximately two-thirds of responding veterinarians had interest in receiving disaster training (22). In addition to Federal Emergency Management Agency (FEMA) Incident Command System training courses available online and various disaster management courses provided by state and local agencies, the National Disaster Life Support Foundation provides two classroom-based courses (Basic Disaster Life Support and Advanced Disaster Life Support) that are designed to train a range of health professionals to respond to and manage casualties from disasters or public health emergencies. To enhance a more focused skill set, the University of Georgia Institute for Disaster Management developed veterinarian-directed BDLS and ADLS courses and an animal/human decontamination course. While these courses provide potential veterinary responders with critical on-scene casualty management skills and understanding of overall scene/incident management, they do little to prepare veterinarians to provide definitive or stabilizing care to human casualties should an event occur. Notably, iterations of the course have been limited due to lack of funding. The gap in preparation identified in 1964 still exists.

It was recommended in 1964 that disaster medical training be given during the veterinary curriculum due to potential difficulty in reaching post-graduate veterinarians stemming from disinterest and competing commitments (10). However, contemporary veterinary students have reported that the current curriculum is already heavy and allows little room for additional requirements; there would likely be little support for inclusion of human-specific disaster medicine (23, 24). Fortunately, the elevated importance of animals in disaster preparation and planning following Hurricane Katrina with the Pet Evacuation and Transportation Standards (PETS) Act and Post-Katrina Emergency Management Reform Act has increased post-graduate veterinarian involvement in disaster management activities. When the United States Department of Agriculture National Veterinary Accreditation Program was revised to align with George W. Bush Administration's Homeland Security Presidential Directives, the role of veterinarians as first responders during certain types of disasters was emphasized (25). If this increased awareness and involvement of veterinarians in disasters can be leveraged, engaging veterinarians in disaster training may not be as difficult as in the past. Also, state veterinary licensing boards could utilize continuing education credits/requirements to further encourage disaster management training. Several options for delivering post-graduate training exist. Options include: 1. A two-part course comprised of an online and in-person training offered through the regional healthcare coalition (if established), 2. A community-based course culminating in a healthcare community-wide exercise developed to provide disaster training and strengthen collaboration among physicians, public health professionals, and veterinarians, 3. A medical university-based course where both didactic sessions and practicums are provided followed by community-level exercises to cement skills and build relationships, 4. A full fellowship with the potential for previous fellows to serve as training ambassadors to their peers in the veterinary field. Even before veterinary school, a sense of commitment to collective health and disaster response could be promoted through a “One Health” -focused pre-professional curriculum; human anatomy and physiology courses would provide a foundation for later training in disaster medical care for humans.

### Legal Limitations

Finally, even if veterinarians are confident in their ability and are willing to provide disaster medical care to humans, the threat of legal consequences after the event may cause veterinarians not to respond. The legal barriers that limit the scope of practice for a veterinarian should be addressed early, and decisions regarding pertinent legislation should be disseminated widely. In Georgia, the Governor has extensive emergency powers including the power to suspend regulatory statutes that would “in any way prevent, hinder, or delay necessary action in coping with the emergency or disaster,” and legal protections are extended to those who act within the Governor's disaster guidance (26). While a disaster significant enough to warrant a declaration of a state of emergency would be clear, the expectation to treat humans and the extent of that scope may not be as evident (27). Practicing medicine without a license is a felony in Georgia, and those convicted face fines of $1000.00 per violation, imprisonment for 2 to 5 years, or both (28). In 1995, civil liability was removed for any person providing services and goods during an emergency, but this particular statute only applies if the provider of the services does not seek or expect compensation (29). Though it may seem callous to consider the financial aspect in light of a disaster, veterinarians are professionals that deserve just compensation for their time and resources in response efforts (30). Legislative efforts should be made to clarify when care can be provided, any limits to the scope of practice, and mechanisms to be reimbursed following resolution of the disaster.

In 2016, the State of Ohio passed a law allowing emergency medical technicians and paramedics to treat injured animals that are found while responding to an emergency. The law allows first responders to provide the following care for animals: maintain airways, give ventilation, administer oxygen, control hemorrhage via direct pressure, stabilize fractures, bandage wounds, and administer certain pharmaceuticals. The law provides immunity from criminal, civil, and professional disciplinary actions as long as care was provided “in good faith in the absence of deliberate misconduct” (31). A similar law was passed in Wisconsin that also allows first responders (to include law enforcement officers) to provide cardiopulmonary resuscitation, bandage wounds, control hemorrhage, and administer oxygen (32). While there are variations in the specifics of the legislation regarding this topic, the overall intent is the same. The laws allow for immediate medical care to a species for which a responder is capable of treating but not licensed to do so. These laws are proof that medical treatment capabilities can be extended to cover additional species and legal protections should be developed to cover emergency and disaster situations.

## Conclusion

As the COVID-19 pandemic began to unfold in 2020, medical experts and multiple state and national governments began to call on veterinarians to supplement the medical response; hundreds of veterinarians responded with a willingness to support response efforts in both clinical and non-clinical roles. Expanded scope of clinical practice activities proposed generally mirror those granted to human first responders for animals; these include airway management/maintenance, intravenous catheter placement and fluid administration, immunization administration, and emergency medical treatment such as hemostasis, bandaging, local anesthesia, and suturing (33–36). While the full extent of veterinary participation in the COVID-19 response remains to be seen, the potential of veterinarians to save human lives has been consistently recorded in the literature over time.

None of the arguments for disaster training for private practice veterinarians should be interpreted as lack of confidence in their collective technical ability to provide emergency medical care for humans; a wide range of clinical skills are expertly practiced by private veterinary practitioners every day around the United States. It is the comfort in translation under distress of this honed clinical acumen to humans that warrants the attention of the disaster management community. The expertise and abilities of veterinarians have been recognized for decades with limited real progress toward enabling them to volunteer to preserve human life in disasters. It is time to address the legal limitations, provide the training, and discuss the ethical dilemmas to enhance the surge capacity of our medical systems. This is especially important in areas where there are already critical provider shortages. Increasing the communication and networking between the healthcare and veterinary communities could improve the response and result in additional lives saved in the event of a mass casualty disaster. While a disaster requiring the coordination of all available medical resources may seem unlikely, the need for veterinary asset integration to the healthcare coalitions with continuous training and exercising cannot be overstated. A highly-trained, ready, and well-resourced health care community prepared for large-scale disasters is in the best interest of all.

## Data Availability Statement

The original contributions presented in the study are included in the article/supplementary material, further inquiries can be directed to the corresponding author/s.

## Author Contributions

LH wrote the initial draft and prepared the published work. RS contributed to concept development and applications. CH and JO'N prepared the published work, specifically with critical reviews, editing, and revisions. All authors contributed to the article and approved the submitted version.

## Conflict of Interest

The authors declare that the research was conducted in the absence of any commercial or financial relationships that could be construed as a potential conflict of interest.

## Publisher's Note

All claims expressed in this article are solely those of the authors and do not necessarily represent those of their affiliated organizations, or those of the publisher, the editors and the reviewers. Any product that may be evaluated in this article, or claim that may be made by its manufacturer, is not guaranteed or endorsed by the publisher.
